# *De Novo* Sequencing, Assembly, and Annotation of Four Threespine Stickleback Genomes Based on Microfluidic Partitioned DNA Libraries

**DOI:** 10.3390/genes10060426

**Published:** 2019-06-03

**Authors:** Daniel Berner, Marius Roesti, Steven Bilobram, Simon K. Chan, Heather Kirk, Pawan Pandoh, Gregory A. Taylor, Yongjun Zhao, Steven J. M. Jones, Jacquelin DeFaveri

**Affiliations:** 1 Department of Environmental Sciences, Zoology, University of Basel, Vesalgasse 1, CH-4051 Basel, Switzerland; 2 Biodiversity Research Centre and Zoology Department, University of British Columbia, Vancouver, BC V6T 1Z4, Canada; marius.roesti@iee.unibe.ch; 3 Institute of Ecology and Evolution, University of Bern, Baltzerstrasse 6, CH-3012 Bern, Switzerland; sichan@bcgsc.ca; 4 Canada’s Michael Smith Genome Sciences Centre at BC Cancer, Vancouver, BC V5Z 4S6, Canada; sbilobram@bcgsc.ca (S.B.); sichan@bcgsc.ca (S.K.C.); hkirk@bcgsc.ca (H.K.); ppandoh@bcgsc.ca (P.P.); gtaylor@bcgsc.ca (G.A.T.); yzhao@bcgsc.ca (Y.Z.); sjones@bcgsc.ca (S.J.M.J.); 5 Department of Medical Genetics, University of British Columbia, Vancouver, BC V6T 1Z3, Canada; 6 Department of Molecular Biology and Biochemistry, Simon Fraser University, Burnaby, BC V5A 1S6, Canada; 7 Faculty of Biological & Environmental Sciences, University of Helsinki, Viikinkaari 1, FI-00014 Helsinki, Finland; jacquelin.defaveri@helsinki.fi

**Keywords:** *Gasterosteus aculeatus*, genome assembly

## Abstract

The threespine stickleback is a geographically widespread and ecologically highly diverse fish that has emerged as a powerful model system for evolutionary genomics and developmental biology. Investigations in this species currently rely on a single high-quality reference genome, but would benefit from the availability of additional, independently sequenced and assembled genomes. We present here the assembly of four new stickleback genomes, based on the sequencing of microfluidic partitioned DNA libraries. The base pair lengths of the four genomes reach 92–101% of the standard reference genome length. Together with their *de novo* gene annotation, these assemblies offer a resource enhancing genomic investigations in stickleback. The genomes and their annotations are available from the Dryad Digital Repository (https://doi.org/10.5061/dryad.113j3h7).

## 1. Introduction

The threespine stickleback (*Gasterosteus aculeatus*) is a small teleost fish widely distributed in both marine and freshwater habitats across the northern hemisphere [1,2]. Because of its ability for colonizing and adapting to diverse types of habitats, the species represents an important system for investigating evolutionary diversification and the underlying genetics [3,4,5]. Such research has been facilitated by the release in 2006 of a high-quality reference genome assembled from Sanger-sequenced plasmids, fosmids, and BAC clones derived from a freshwater individual from Alaska [6]. This genome, hereafter called the “reference genome”, has a total ungapped size of 447 Mb, of which 95% has been anchored to the 21 chromosomes through three rounds of re-assembly of the original sequence scaffolds or contigs [7,8,9]. Despite the high quality of this resource, access to additional, independently sequenced and assembled stickleback genomes appears desirable, given the wide geographic distribution of the species, and the extensive population structure and phenotypic and genetic diversity exhibited across its range. In this paper, we report the generation and annotation of four *de novo* stickleback genome assemblies.

## 2. Methods and Materials

### 2.1. Stickleback Samples, DNA Library Preparation, Sequencing

Populations were chosen to mirror the species’ wide ecological diversity and geographic distribution. Specifically, we selected a marine (anadromous) and a freshwater-resident population from both the Atlantic (SYL, NID) and the Pacific region (BAM, BOT) (Table 1). From each site, a single individual was sampled with unbaited minnow traps on breeding grounds during the spring of 2016, except for the SYL individual sampled from a laboratory line in the same year. After euthanasia according to standard protocols, the focal individuals were immediately frozen at −20 °C or −80 °C to minimize DNA degradation. To facilitate the assembly of chromosome 19, which is the sex chromosome heterogametic (XY) in threespine stickleback males [10], we considered only female individuals.

To obtain high molecular weight DNA, we slowly thawed the specimens, immediately sampled 50 mg of liver (SYL, BAM, BOT) or muscle (NID) tissue, and performed extractions using the QIAGEN MagAttract high molecular weight DNA Kit (Qiagen, Germantown, MD, USA), following the manufacturer’s protocol. DNA integrity was assessed using pulsed-field gel electrophoresis (PFGE; the resulting gel images are provided as Figure S1 in the Supplementary Materials). The DNA obtained (predominantly <50 kb) was then used without size selection to generate microfluidic partitioned libraries using the Chromium System (10x Genomics Inc., Pleasanton, CA, USA). Details on DNA quality assessment and the Chromium library preparation protocol are specified in [11]. For the individuals SYL and NID, only a single Chromium library was generated, while for BAM and BOT, two replicate libraries were produced. These six total DNA libraries were then barcoded individually, pooled to equal total molarity among individuals, and paired-end sequenced to 150 base pairs (bp) in three lanes of an Illumina HiSeq X instrument. This produced between 504 and 542 million raw sequence reads per individual in total. The raw sequence data are available at the NCBI Short Read Archive (SRA) under the BioProject accession number PRJNA525775 (the genome assemblies and their annotations are, in addition to Dryad, also available under the same NCBI BioProject number).

### 2.2. Genome Assembly and Annotation

As a first step, we filtered reagent sequences (approximately 1%) from the raw paired-end reads. Then we performed genomic assemblies by using the proprietary Supernova assembler (10x Genomics, San Francisco, CA, USA) with default parameters on a 750 Gb server, running the jobs serially to avoid resource conflict. To maximize assembly quality, we explored different combinations of assembler versions (1.20 and 2.01) and sequence coverages (from approximately 30× to 145×). For NID, the highest quality, as judged by N50 values and total assembly length, was achieved with Supernova version 2.01, while for the other individuals, version 1.20 performed best. Moreover, for all four stickleback individuals, optimal sequence coverage was above the maximum of 56× recommended for Supernova assembly of human genomes (89×, 142×, 145×, and 77× for SYL, NID, BAM, and BOT, respectively). An effort to further increase contiguity by using ARCS [15] did not improve the quality of the assemblies; hence, our genomes represent the assemblies obtained by Supernova, with scaffolds smaller than 1 kb excluded. No attempt was made to arrange our assembled scaffolds to the level of physical chromosomes.

Each of the four assemblies was then annotated for protein coding genes by using the MAKER platform (version 2.31.9; Yandell Lab, Salt Lake City, UT, USA). MAKER produces a single set of annotated genes by combining the ab initio gene prediction from three programs (AUGUSTUS [16], Snap [17], GeneMark [18]), informed by experimental gene evidence. As gene evidence, we used the threespine stickleback cDNA underlying the annotation of the reference genome (27,628 transcripts; available on Ensembl), and 538,010 protein sequences included in Swiss-Prot. AUGUSTUS predictions were trained on zebrafish genes, SNAP was trained using the 2586 highly conserved vertebrate genes predicted by BUSCO (version 2.0.1) [19,20], while GeneMark was self-trained.

### 2.3. Comparative Sequence Alignment

To illustrate the value of the new assemblies as resources for sequence alignment, we used a sample of 5 million 150 bp paired-end reads produced by Illumina HiSeq2500 whole-genome sequencing of pooled DNA from 72 field-caught stickleback from the NID population (the individuals derive from an experimental study [21]; sequence data: Laurentino and Berner, manuscript in preparation). These reads were paired-end aligned with Novoalign (version 3.00; http://www.novocraft.com/products/novoalign) to the reference genome and to each of the four new assemblies, each time using identical alignment parameters (main settings: –t540, –g40, –x12). The observed proportion of unique alignment to each assembly was standardized by the alignment proportion observed when using the reference genome. The reason for this standardization was that we were not primarily interested in the absolute alignment success, but in how alignment success compared among the assemblies to which the same set of reads was matched.

Since the NID population is located in the Atlantic part of the species’ range, we repeated the above alignment protocol by using an analogous sample of whole-genome sequence reads generated in a similar way from a pool of DNA from 62 stickleback individuals from a Pacific freshwater population (Misty Lake, Vancouver Island, Canada [22,23]; sequence data: Haenel and Berner, manuscript in preparation).

## 3. Results and Discussion

**Figure 1:**
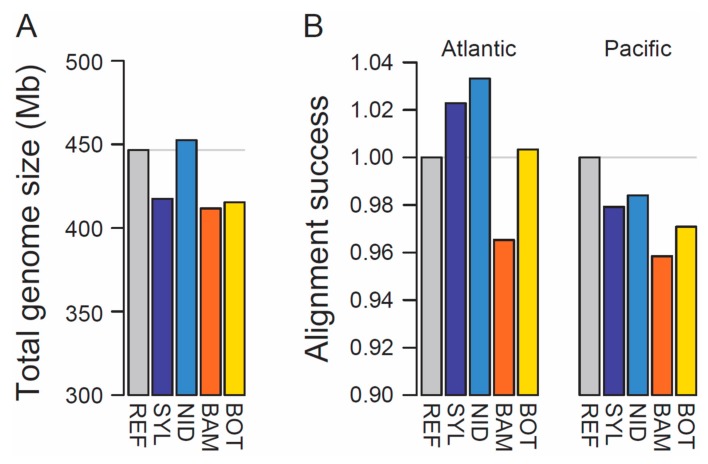
(**A**) Total ungapped length of the reference (REF) stickleback genome and the four new assemblies (SYL, Atlantic marine; NID, Atlantic freshwater; BAM, Pacific marine; BOT, Pacific freshwater). (**B**) Alignment success of whole-genome sequence reads from an Atlantic (left, NID) and a Pacific (right, Misty Lake) freshwater population when matched to each of the five assemblies, standardized by the alignment success achieved with the reference genome. Note that in both (**A**) and (**B**), the *y*-axis is strongly truncated to increase visual resolution in the upper range of the scale. The gray horizontal lines indicate the values for the reference genome.

The four new stickleback genome assemblies varied in length from 412 to 453 Mb (Table 1), thus ranging from approximately 8% smaller to 1% larger than the reference genome (Figure 1A). Contiguity was modest and highly variable, with scaffold numbers varying 2.5-fold and N50 values varying 12-fold among the individuals (Table 1). Interestingly, the assemblies based on two replicate Chromium libraries (BAM, BOT) were not superior in completeness or contiguity to those derived from a single library. This leads us to speculate that molecular weight of the extracted DNA—which was highest in the NID individual (details not presented)—may be a more critical determinant of assembly quality than the number of libraries when using the Chromium system. We also found no indication of a correlation among individuals between total read number and total assembly length.

The average number of genes annotated was around 18,000 across the assemblies (genes with evidence; Table 1). Annotated gene number was strongly correlated with total assembly length (r = 0.97), indicating that the sequences missing in the less complete assemblies were not unusual with respect to gene content.

Aligning whole-genome sequence reads from the NID population with identical alignment parameters to all genomes revealed highest success when matched to SYL and especially NID—the two Atlantic assemblies (Figure 1B, left). This result cannot be attributed to genome completeness alone because the SYL genome is relatively incomplete, but must reflect substantial overall sequence divergence between Atlantic and Pacific stickleback. Consistent with this view, when aligning whole-genome reads from a Pacific population (Misty Lake) to all genomes, the success of alignment to the Atlantic assemblies dropped below the success observed for the reference genome (Figure 1B, right). Our comparative alignment analysis thus highlights the potential of our new assemblies, particularly the Atlantic ones, to complement population genomic analyses based on the reference genome. We further anticipate that our new resources will facilitate primer design for applications like targeted sequencing and genome editing, and the identification of structural variation within the stickleback genome.

## Figures and Tables

**Table 1:** Characterization of the four stickleback individuals and their genome assemblies.

Assembly	SYL	NID	BAM	BOT
Region	Atlantic	Atlantic	Pacific	Pacific
Habitat type	Marine	Freshwater	Marine	Freshwater
Locality [Reference]	List, Sylt, Germany	Aach stream, Switzerland [12]	Bamfield Inlet, Vancouver Island, Canada [13]	Boot Lake, Vancouver Island, Canada [14]
Geographic coordinates	55°01′49.04″ N, 8°25′37″ E	47°33′29.25″ N, 9°16′42.38″ E	48°49′12.69″ N, 125°8′57.9″ W	50°03′00.2″ N, 125°32′27.4″ W
Number of scaffolds	15,853	10,246	25,430	18,433
N50 (Mb)	0.396	3.636	0.446	0.307
Longest scaffold (Mb)	3.12	16.02	4.33	3.81
Total assembly length (Mb) (gapped length in parentheses)	417.5 (431.8)	452.5 (467.5)	411.7 (445.7)	414.9 (427.3)
Number of annotated genes with experimental evidence	18,513	19,928	17,789	18,413

## Supplementary Materials

The following is available online at https://www.mdpi.com/2073-4425/10/6/426/s1, Figure S1: DNA electrophoresis gel image.
