# Supplementary material for: De Novo Sequencing, Assembly, and Annotation of Four Threespine Stickleback Genomes Based on Microfluidic Partitioned DNA Libraries
Source: Genes (Basel). 2019 Jun 3;10(6):426. doi: 10.3390/genes10060426 (PMC6627416; doi:10.3390/genes10060426)
Supplement: Supplementary file 1 [file genes-10-00426-s001.pdf]

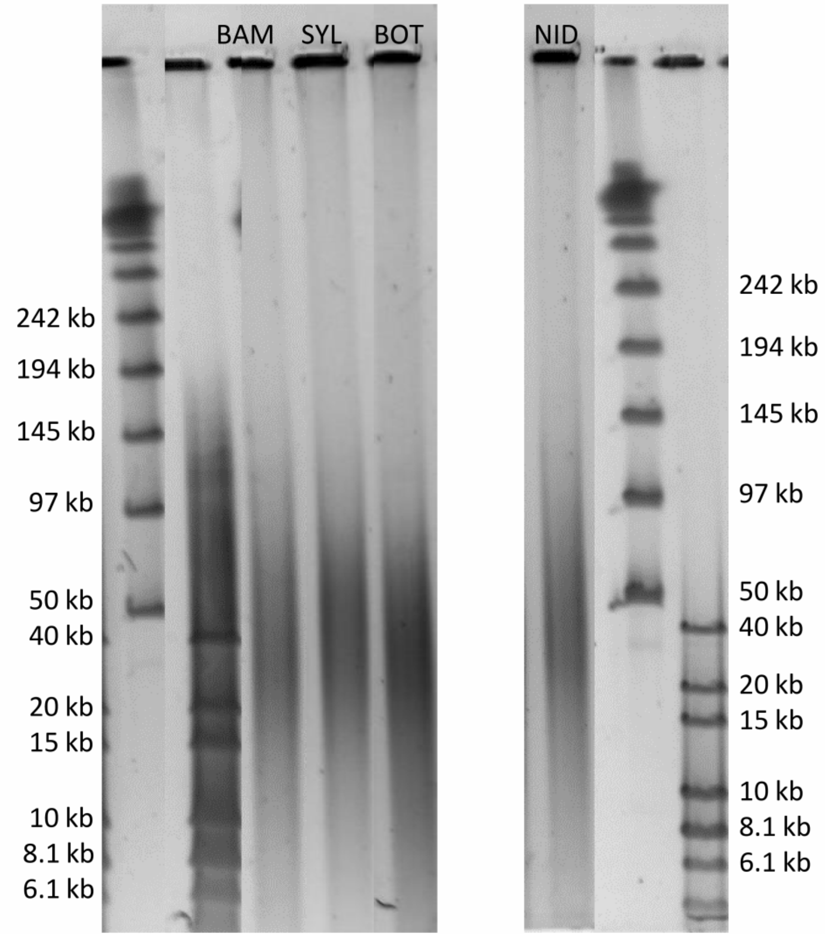

Figure S1. Integrity of the DNA of the four individuals used for *de novo* genome sequencing and assembly, visualized by pulsed-field electrophoresis gels. The first and last two lanes are reference ladders.
